# Loss of the ER membrane protein complex subunit *Emc3* leads to retinal bipolar cell degeneration in aged mice

**DOI:** 10.1371/journal.pone.0238435

**Published:** 2020-09-04

**Authors:** Xiong Zhu, Xin Qi, Yeming Yang, Wanli Tian, Wenjing Liu, Zhilin Jiang, Shuzhen Li, Xianjun Zhu

**Affiliations:** 1 Prenatal Diagnosis Center, Sichuan Provincial Key Laboratory for Human Disease Gene Study, Sichuan Provincial People’s Hospital, School of Medicine, University of Electronic Science and Technology of China, Chengdu, Sichuan, China; 2 Neurosurgery Research Laboratory, West China Hospital, Sichuan University, Chengdu, Sichuan, China; 3 Department of Ophthalmology, Shangqiu First People’s Hospital, Shangqiu, Henan, China; National Eye Institute, UNITED STATES

## Abstract

The endoplasmic reticulum (ER) membrane protein complex (EMC) is a conserved protein complex involved in inserting the transmembrane domain of membrane proteins into membranes in the ER. EMC3 is an essential component of EMC and is important for rhodopsin synthesis in photoreceptor cells. However, the in vivo function of *Emc3* in bipolar cells (BCs) has not been determined. To explore the role of *Emc3* in BCs, we generated a BC-specific *Emc3* knockout mouse model (named *Emc3* cKO) using the Purkinje cell protein 2 (Pcp2) Cre line. Although normal electroretinography (ERG) b-waves were observed in *Emc3* cKO mice at 6 months of age, *Emc3* cKO mice exhibited reduced b-wave amplitudes at 12 months of age, as determined by scotopic and photopic ERG, and progressive death of BCs, whereas the ERG a-wave amplitudes were preserved. PKCa staining of retinal cryosections from *Emc3* cKO mice revealed death of rod BCs. Loss of *Emc3* led to the presence of the synaptic protein mGLuR6 in the outer nuclear layer (ONL). Immunostaining analysis of presynaptic protein postsynaptic density protein 95 (PSD95) revealed rod terminals retracted to the ONL in *Emc3* cKO mice at 12 months of age. In addition, deletion of *Emc3* resulted in elevated glial fibrillary acidic protein, indicating reactive gliosis in the retina. Our data demonstrate that loss of *Emc3* in BCs leads to decreased ERG response, increased astrogliosis and disruption of the retinal inner nuclear layer in mice of 12 months of age. Taken together, our studies indicate that *Emc3* is not required for the development of BCs but is important for long-term survival of BCs.

## Introduction

The ER membrane protein complex (EMC) is a highly conserved complex that was first identified in a genetic screen for the accumulation of misfolded membrane proteins in yeast [1]. This complex was then found to interact with the ER-associated degradation pathway, indicating functions in the quality control process of transmembrane proteins [2–4]. In recent years, the EMC has been shown to play important roles in multiple cellular events, such as viral infection, autophagy, lipid transfer, vision development and lung disease [5–13]. The EMC functions as an ER chaperone for multipass transmembrane proteins, as well as an insertase for selective tail-anchored membrane proteins [14–16]. Mutation of the zebrafish *emc3* homolog, *partial optokinetic response b* (*pob*), causes degeneration of photoreceptor cells, and *Drosophila emc3* is required for the biosynthesis and transport of rhodopsins [17,18]. Loss of *Emc3* in mouse photoreceptor cells leads to mislocalized rhodopsin and degeneration of photoreceptor cells [19]. However, the role of *Emc3* in BCs has not been addressed.

In this study, we investigated the roles of *Emc3* in BCs by generating *Emc3* BC-specific conditional knockout mice (cKO). Loss of *Emc3* in BCs results in a decrease in b-wave amplitude, as shown by scotopic and photopic ERG tests and degeneration of BCs, whereas the a-wave responses were preserved in aged mice. Astrogliosis was prominent in *Emc3* cKO retinas at 12 months of age. Our data demonstrate the importance of *Emc3* in the long-term survival of BCs.

## Materials and methods

### Generation of *Emc3* BC knockout mice

All experimental procedures were performed following an approved research protocol, which was reviewed and approved by the Institutional Animal Care and Use Committee of Sichuan Provincial People’s Hospital (2104NSF(09)). All methods were carried out in accordance with relevant guidelines and regulations. Mice were raised under a 12 h light-12 h dark cycle.

The *Emc3* conditional knockout allele (*Emc3*^*fl/+*^) was described previously [19]. BC-specific *Emc3* knockout mice were generated by mating *Emc3*^*fl/+*^ mice to transgenic Cre line Pcp2-Cre [20]. The F1 progeny *Emc3*^*fl/+*^; Pcp2-Cre mice were crossed to generate *Emc3*^*fl/fl*^ mice (*Emc3*^*fl/fl*^; Pcp2-Cre, named *Emc3* cKO). *Emc3*^*fl/fl*^, *Emc3*^*fl/+*^ or *Emc3*^*fl/+*^; Pcp2-Cre mice were used as controls. To monitor the efficiency of Cre-mediated deletion of the floxed exon, a tdTomato reporter was used (Jackson Laboratory Cat# JAX:007914).

Tissues were harvested after animals were euthanized by CO_2_ exposure, which was confirmed by decapitation. No animals were excluded from this study. The number of mice used in each test was listed in S2 Table.

## ERG recordings

ERG readings were recorded in live mice of both sexes as described previously [19, 21, 22] using an Espion Visual Electrophysiology System from Diagnosis, LLC (Littleton, MA, USA). Briefly, mice were dark-adapted overnight, and dim red light was used in all subsequent procedures. Mice were anaesthetized with a mixture of ketamine (16 mg/kg body weight) and xylazine (80 mg/kg body weight) in sterile saline. Their eyes were dilated with tropicamide and phenylephrine, and tetracaine (0.5%) was applied before the ERG test. Mouse body temperatures were maintained at 37°C with a heating platform. After pupil dilation, the scotopic and photopic ERG responses were recorded. Dark-adapted ERGs were recorded using flashes with intensities ranging from 0.003 to 20 cd·s/m^2^. Cone-mediated ERGs were recorded with white flashes after 20 min of complete light adaptation. The total amplitudes of the oscillatory potentials (OPs) were quantified by measuring the amplitude of each wavelet and adding them together. The amplitude of the a-wave was calculated from the baseline to 8 ms immediately after the flash. The amplitude of the b-wave was calculated from the baseline to the positive peak for low-luminance stimuli. For high-luminance stimuli, the b-wave amplitude was measured from the negative a-wave trough to the b-wave peak following the high-frequency oscillatory potentials. Similarly, the amplitude of the cone b-wave was calculated from the initial negative trough to the b-wave peak. The total amplitudes of the OPs were quantified by measuring the amplitude of each wavelet and adding them together.

### Reverse transcription polymerase chain reaction (RT-PCR)

Tissues were dissected and placed into RNAlater (Ambion, Austin, TX, USA) at room temperature. Total RNA was prepared from these tissues using TRIzol reagent (Life Technologies) according to the manufacturer’s instructions. RNA samples were treated with RNase-free DNase I (Ambion) to remove genomic DNA, and the RNA concentration was determined with a NanoDrop (ND-1000) spectrophotometer. A total of 3 μg of RNA was reverse transcribed using random primers and a MessageSensor RT kit (Ambion, TX, USA). The primers used for RT-PCR of mouse genes are listed in S1 Table of the supplementary data. PCR was performed with Taq polymerase (New England Biolabs, MA, USA), and the PCR products were resolved on 3% agarose gels.

## Histology and immunohistochemistry

For histological staining with hematoxylin and eosin (H&E), eyes were enucleated from *Emc3* cKO and control mice and incubated overnight at 4°C in 1.2% glutaraldehyde and 0.8% paraformaldehyde (wt/vol) in 0.1 M phosphate buffer (PB, pH 7.4). The eyes were then washed in PB and embedded in paraffin. Five-micrometer sections were cut, stained and scanned with a slide scanner. H&E stained sections were used to evaluate the thickness of the outer nuclear layer (ONL) and inner nuclear layer (INL). Samples were measured by a technician who was blinded to the experimental groups.

For immunostaining analysis, enucleated eyes were fixed in fresh 4% paraformaldehyde (wt/vol) in 0.1 M phosphate buffer (PB, pH 7.4). After rinsing, eyes were cryoprotected by treatment with a sucrose series (10%, 15%, and 30% in PB) and frozen in optimal cutting temperature compound (VWR, Radnor, PA, USA, catalog #4583). Ten-micrometer sections were cut on a freezing microtome and then were stained as previously described [19]. Primary antibodies were diluted in blocking solution and incubated on retinal sections overnight at room temperature. Antibodies used in this study were as follows: rabbit anti-EMC3 (1:500, #702736, Invitrogen, Waltham, MA, USA), mouse anti-PKCa (1:300; Sigma-Aldrich, St. Louis, MO, USA), rabbit anti-PSD95 (1:500; Chemicon, Temecula, CA), rabbit anti-mGluR6 (1:100; # PA1-32783, Invitrogen) and rabbit anti-glial fibrillary acidic protein (GFAP) (1:300; catalog #12389, Cell Signaling Technology, Danvers, MA, USA). Secondary antibodies were ordered from Invitrogen (goat anti-mouse, -rabbit or -rat IgG labeled with Alexa Fluor 488, Alexa Fluor 568, Alexa Fluor 594 or Alexa Fluor 647). Images were captured on a laser scanning confocal microscope (LSM800) (Zeiss, Thornwood, NY, USA). ImageJ software (NIH, Montgomery, MD, USA) was used for image analysis.

## Western blotting

Mouse retinas were lysed and homogenized in lysis buffer (50 mM Tris-HCl, 150 mM NaCl, 1% Triton X-100, 0.5% sodium deoxycholate, 0.1% sodium dodecyl sulfate, pH = 7.4, supplemented with cOmplete™ Protease Inhibitor Cocktail (11697498001, Roche, Redwood City, CA, USA)). Homogenates were cleared by centrifugation at 15,000 *g* for 20 min at 4°C. Protein concentrations were determined by BCA protein assay reagent (NCI3227CH, Pierce, Thermo Fisher Scientific, Pittsburgh, PA, USA). Equal amounts of protein samples were separated by SDS-PAGE and transferred to PVDF membranes, and then immunoblotting was performed as described previously [23]. ImageJ was used to calculate the relative density of the protein. At least three independent western blots were performed, and one typical blot from each experiment is shown.

## Statistical analysis

Unless otherwise stated in the text and figure legends, the data are presented as the mean ± SEM. The data sets were tested for normal distribution using Kolmogorov–Smirnov test. If the data set is not normally distributed, non-parametric statistic is used. ANOVA tests were performed for ERG datasets in Figs 3 and 4 and the INL thickness measurement dataset in Fig 6. Post-hoc tests were performed if a significant ANOVA is achieved. All statistical tests are described in the corresponding figure legends, and Prism (Prism 7.0 software; GraphPad Software, Inc., La Jolla, CA, USA) was used. In all experiments, "n" indicates the number of animals used. The total number of animals used in each experiment is also reported in the figure legends and in S2 Table. Asterisks in the figures indicate the following p values: *≤0.05, **≤0.01, and ***≤0.005.

## Results

### Deletion of *Emc3* in bipolar cells causes reduced ERG b-waves and death of bipolar cells in aged mice

**Fig 1:**
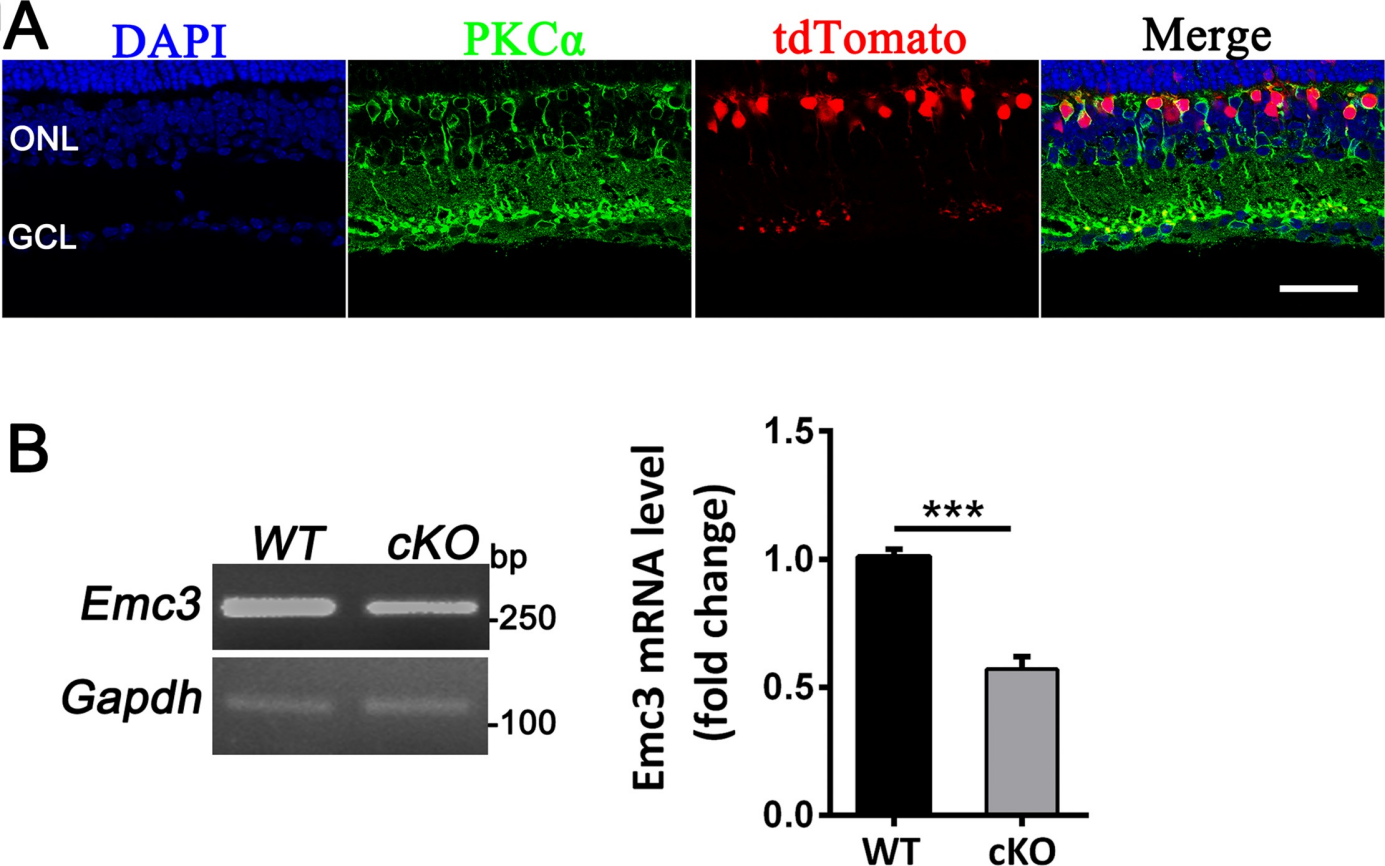
*Emc3* expression levels were reduced in *Emc3* cKO mice. (A) Verification of Pcp2-Cre specification using tdTomato reporter mice. Td-Tomato reporter mice were crossed with Pcp2-Cre mice, and Cre expression was monitored (red). Rod bipolar cells were stained with PKCα (green). Nuclei were stained with DAPI (blue). Td-tomato-expressing cells were significantly associated with rod bipolar cells, indicating specific expression of Pcp2-Cre. The sample size was n = 4 for both control and *Emc3* cKO mice. (B) RT-PCR analysis showed a 45% reduction in *Emc3* expression in *Emc3* cKO mice compared to that of controls. A t-test was performed. The sample size was n = 4 for both control and *Emc3* cKO mice. *** p < 0.001; Error bars represent SD.

The EMC subunit *Emc3* is expressed in the retina and was previously shown to play essential roles in the synthesis of rhodopsin in retinal photoreceptor cells [19]. *Emc3* is expressed in the BCs (S1 Fig). However, its function in BCs has not been determined. We generated a retinal bipolar cell-specific *Emc3* knockout model (S2 Fig) to investigate whether deletion of *Emc3* affects the function and survival of these cells using Pcp2 transgenic line, which drives Cre expression in approximately 75% of rod bipolar cells and type 2 and 6 ON cone bipolar cells [24, 25]. A tdTomato reporter was used to monitor the efficiency of Cre-mediated deletion of the floxed exon (Fig 1A) [26]. The majority of the tdTomato-expressing cells were rod bipolar cells positive for the rod bipolar cell marker PKCα. This result is similar to the expression pattern (75% of the tdTomato-expressing bipolar cells were positive for PKCα) reported by Lu et al. [24] (Fig 1A). Some ON cone bipolar cells were also positive for tdTomato labeling (Fig 1A). *Emc3* cKO mice were genotyped by PCR (S3 Fig) and were born at a typical Mendelian ratio. RT-PCR analysis of cDNA extracted from *Emc3* cKO mice revealed that *Emc3* expression decreased by 40% (Fig 1B).

**Fig 2:**
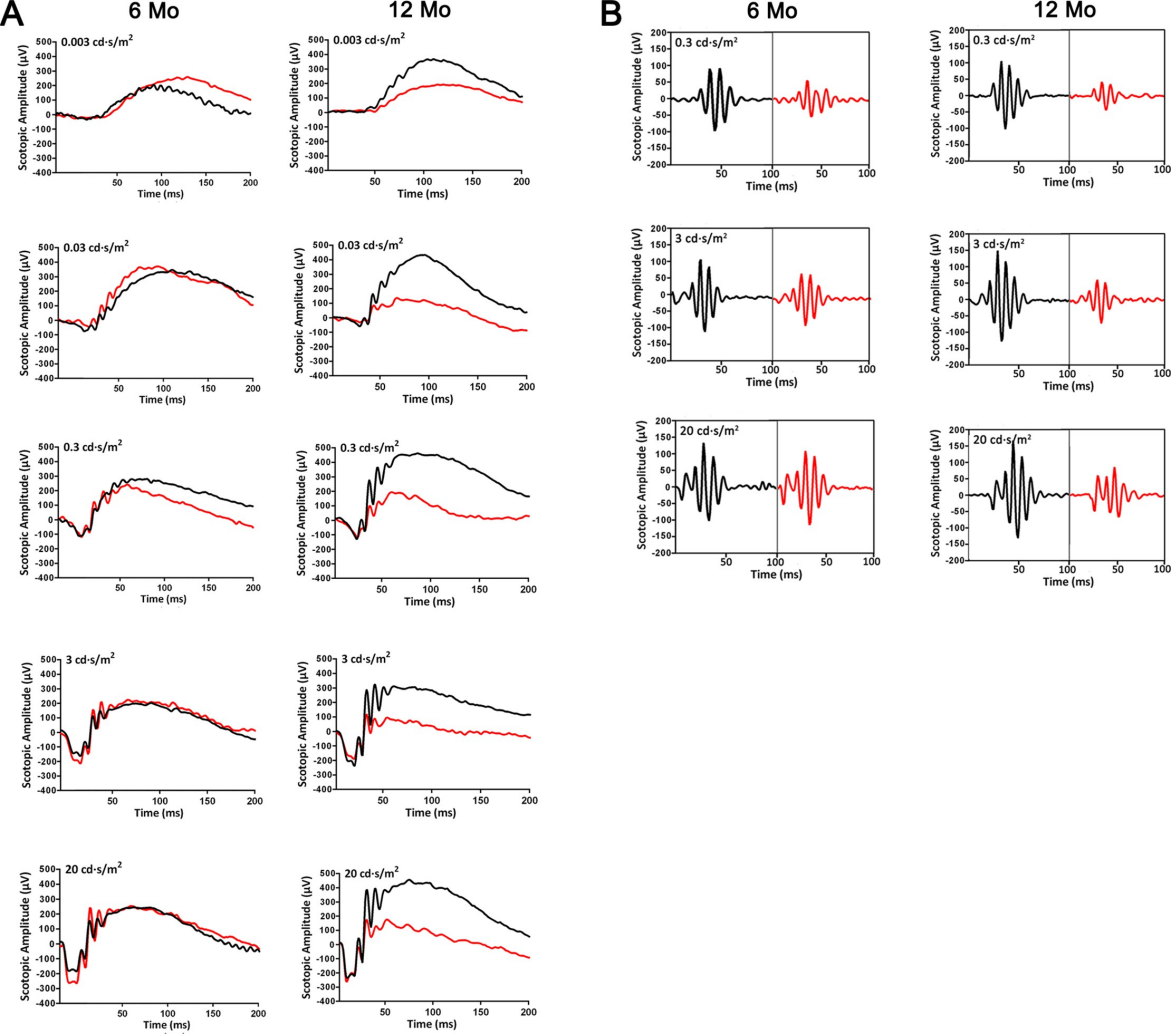
Representative ERG test trace in *Emc3* cKO mice at 6 and 12 months of age. **(A)** Representative electroretinogram (ERG) traces corresponding to responses elicited by scotopic conditions at flash intensities from 0.003 to 20 cd sec/m^2^ in mice at 6 months and 12 months of age. **(B)** OP peak amplitudes under scotopic reaction conditions with a flash intensity of 0.003 to 20 cd sec/m^2^ in mice at 6 months and 12-months of age.

**Fig 3:**
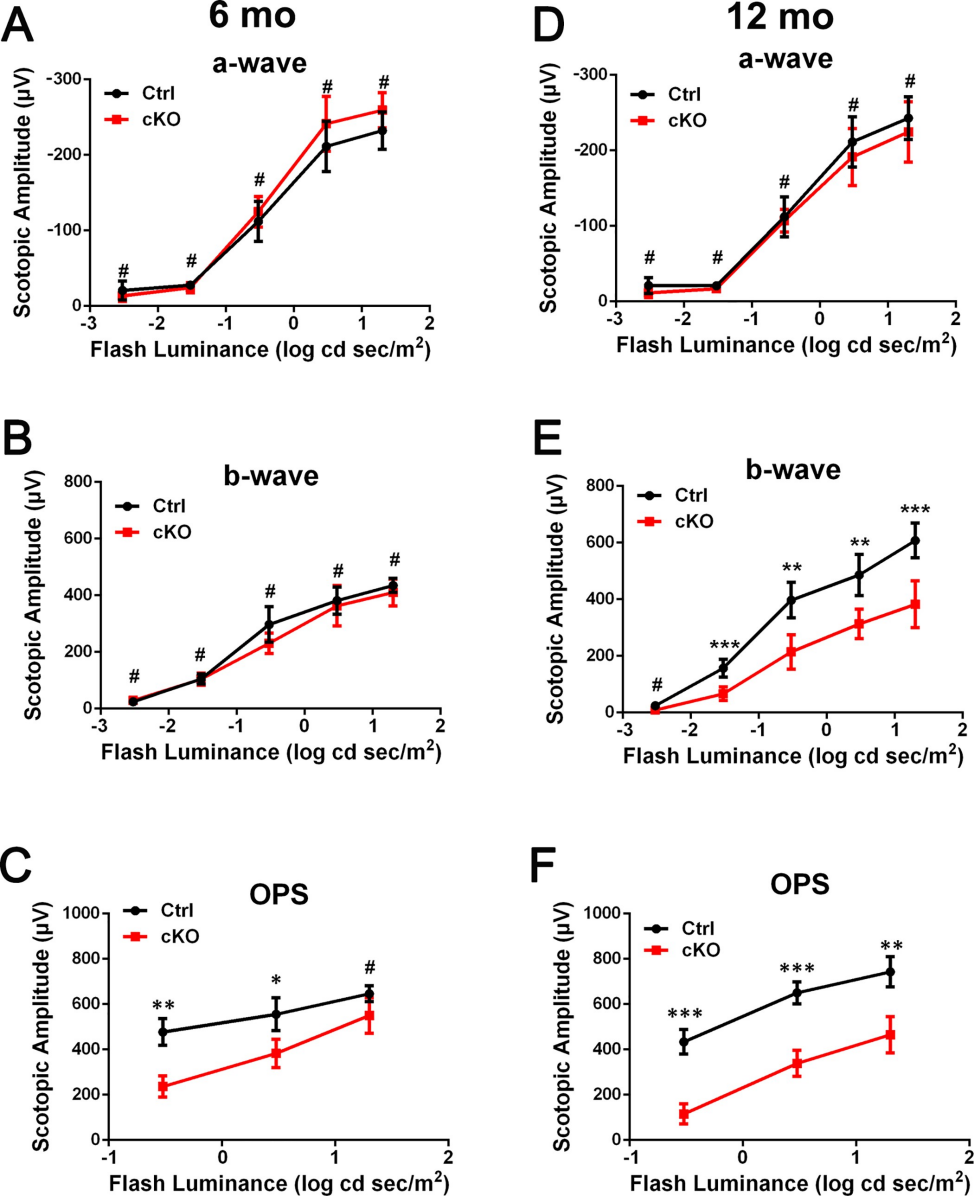
Reduced scotopic ERG b-wave amplitudes in *Emc3* cKO mice at 12 months of age. ANOVA tests were performed for the amplitudes of a-wave, b-wave and OPs of mice at 6 months and 12 months of age. Post-hoc tests were performed for b-wave and OPs. At 6 months of age, there was no significant difference in the amplitude of the scotopic a-wave and b-wave. However, at 12 months of age, the amplitudes of the photopic and scotopic ERG b-waves were significantly reduced, while the amplitudes of the a-wave were preserved. The OP values of the *Emc3* cKO group were lower than those of the control group. The sample size was n = 4 for both the control and cKO groups. * * p < 0.01; * * * p < 0.001. Error bars represent SD.

**Fig 4:**
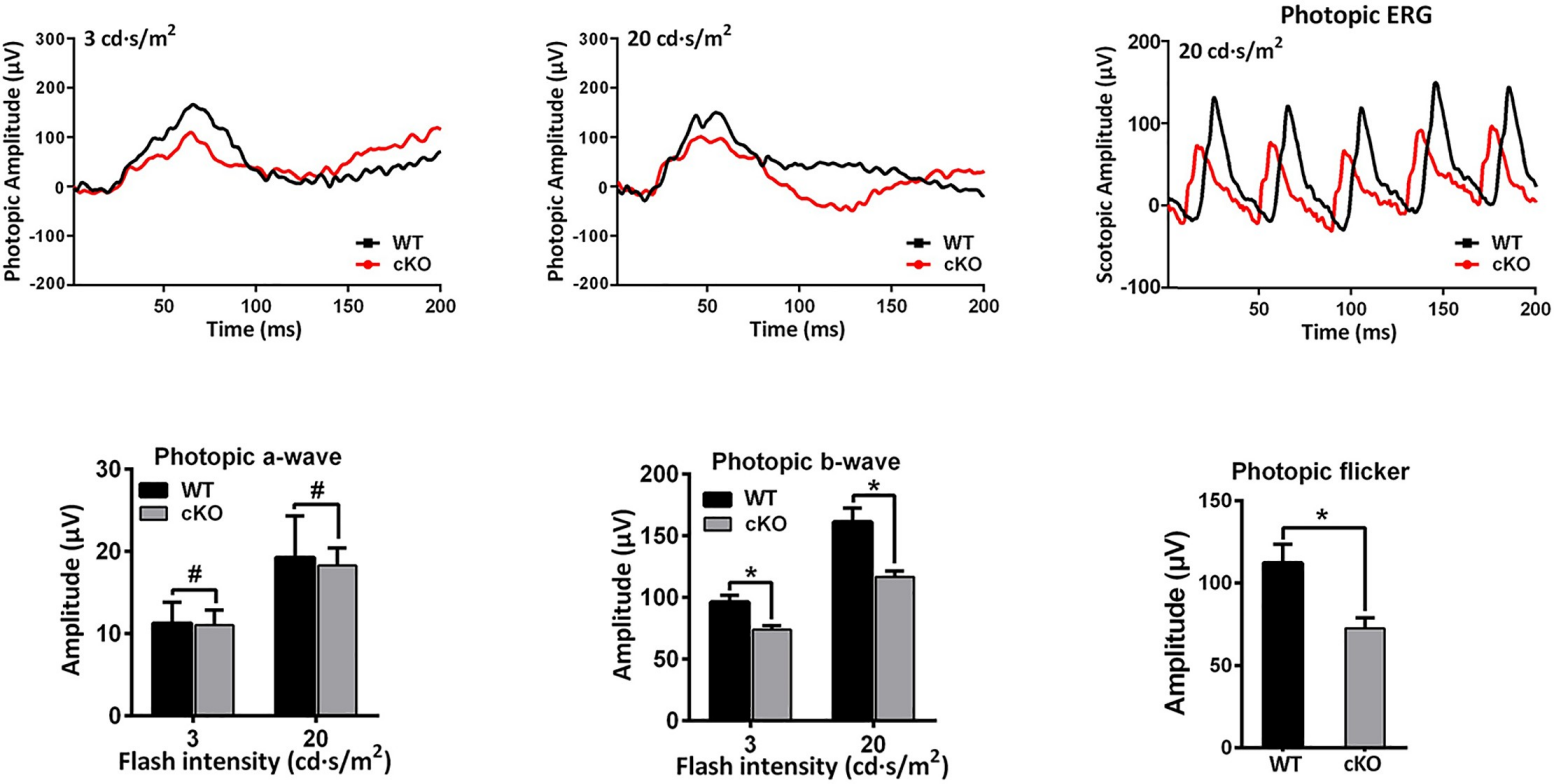
Reduced photopic ERG b-wave amplitudes in *Emc3* cKO mice at 12 months of age. Representative electroretinograms (ERG) traces corresponding to responses elicited by photopic conditions at flash intensities from 0.3 and 20 cd sec/m^2^ in mice at 12 months of age. ANOVA tests were performed for the amplitudes of b-wave at 12 months of age. A post-hoc test was performed. T-test was performed for photopic flicker. * * p < 0.01; * p < 0.05. Error bars represent SD.

To evaluate the physiological functions of *Emc3* in retinas, ERG analysis was performed on control and *Emc3* cKO mice at 6 and 12 months of age. At 6 months of age, under scotopic conditions, no visible changes were observed for the a-wave and b-wave amplitudes in *Emc3* cKO mice (Fig 2A, left panels; Fig 3A and 3B). However, at 12 months of age, the scotopic b-wave amplitude was reduced by 50% at 0.3, 3, and 20 cd sec/m^2^ luminance, and the photopic b-wave amplitude was also reduced by 30% at both 3 and 20 cd sec/m^2^ flash intensity (Fig 3) in *Emc3* cKO mice (Fig 2A, right panels; Fig 3E); the a-wave amplitude did not change (Fig 2A, right panels; Fig 3B), indicating postreceptoral defective visual transmission in the inner retina. In addition, the total amplitude of OPs, which is an indicator of the sensitivity of the inner retina [27], decreased to 51%, 62%, and 61% in *Emc3* cKO mice at 0.3, 3 and 20 cds/m^2^ flash intensity, respectively, in 12-month-old *Emc3* cKO **mice** (Figs 2B and 3F). A decrease in the amplitude of the OPs, which represents activity in synaptic inhibition feedback neural pathways in the inner retina, which includes bipolar cells and ganglion cells, provides additional evidence that postreceptoral transmission in the retina is impaired. Since Pcp2-Cre is also expressed in cone bipolar cells [24], as revealed by red tomato-positive cells not stained by a PKCα antibody (Fig 1A), we examined the ERG response under photopic conditions. Under photopic conditions, the b-wave amplitude was reduced by 30% at both 3 and 20 cds/m^2^ flash intensities in *Emc3* cKO mice (Fig 4). In summary, our ERG data indicate that the loss of *Emc3* significantly impairs visual transmission in the inner retina at 12 months of age.

**Fig 5:**
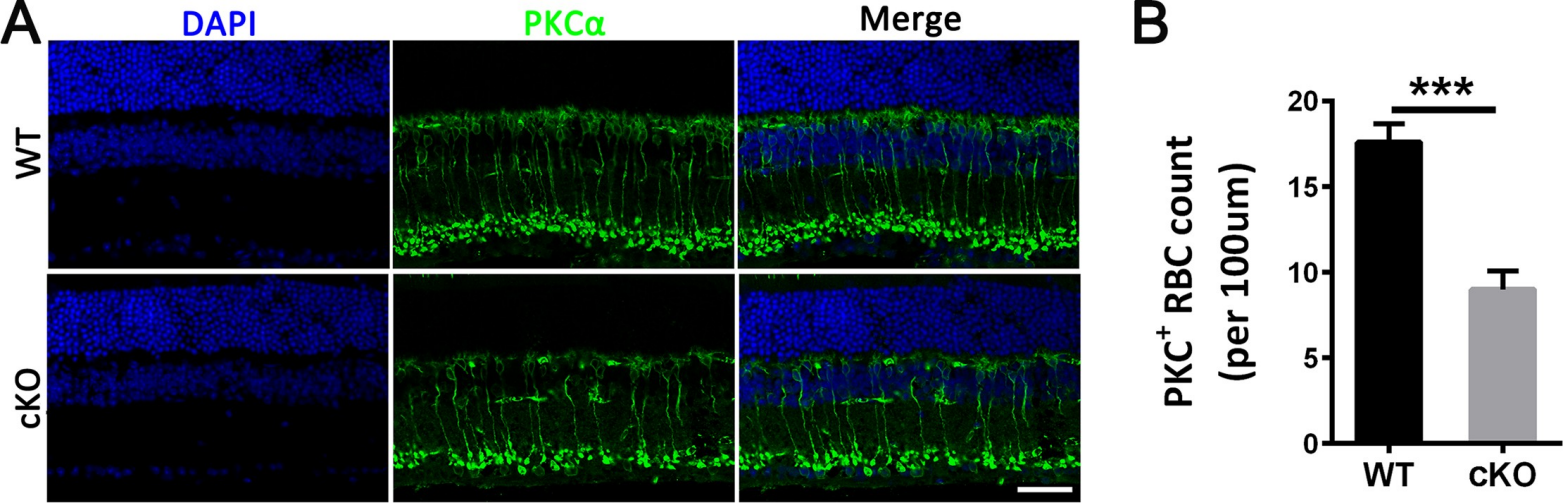
*Emc3* cKO mice exhibited degeneration of rod bipolar cells. **(A)** Representative immunostaining of retinal sections of control (WT) and *Emc3* cKO retinas at 12 months old using anti-PKCα (green). Nuclei were counterstained with DAPI (blue). Scale bar, 25 μm. **(B)** Quantification of PKCα-positive cells per 100 μm of section in the same position. A t-test was performed. The number of PKCα-positive cells clearly declined in 12-month-old cKO mice. The sample size was n = 6 for both WT and cKO mice. n = number of independent biological replicates. * ** p < 0.001; Error bars represent SD.

**Fig 6:**
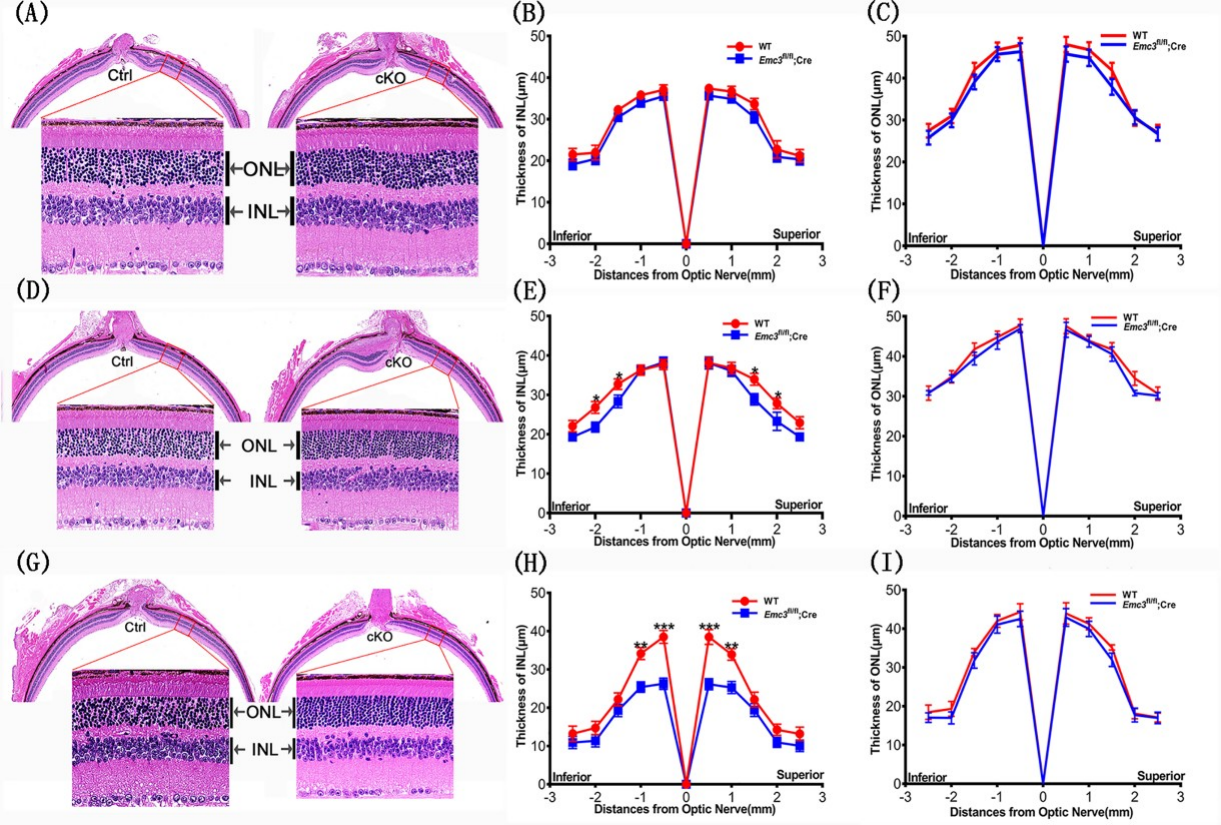
Progressive degeneration of bipolar cells. (A) No visible degeneration in *Emc3* cKO mice at 3 months of age. Representative H&E stained retinal sections of control and *Emc3* cKO mice at 3 months of age, oriented with the inferior pole to the left and superior pole to the right. High magnification images are displayed on the lower panel of each image. Scale bar, 25 μm. The sample size was n = 4 for both controls and *Emc3* cKO mice. (B and C) Quantification of inner nuclear layer (INL) and outer nuclear layer (ONL) thickness at specified distances from the optic nerve head of the control and *Emc3* cKO retina. No difference was observed in the thickness of the INL and ONL when comparing control and *Emc3* cKO mice. (D) Degeneration in middle retinas in *Emc3* cKO mice at 6 months of age. Representative H&E stained retinal sections of control and *Emc3* cKO mice at 6 months of age, oriented with the inferior pole to the left and superior pole to the right. High magnification images are displayed on the lower panel of each image. Scale bar, 25 μm. The sample size was n = 4 for both control and *Emc3* cKO mice. (E and F) Quantification of inner nuclear layer (INL) and outer nuclear layer (ONL) thickness at specified distances from the optic nerve head of the control and *Emc3* cKO retina. The thickness of the INL was reduced in *Emc3* cKO mice. Two-tailed t-test. *, p<0.05. (G) Representative H&E stained retinal sections of control (WT) and *Emc3* cKO retinas in mice at 12 months of age, oriented with the inferior pole to the left and superior pole to the right. Higher magnification images are shown in the lower panel of each image. Scale bar, 25 μm. (H and I) Quantification of the outer nuclear layer (ONL) (D) and inner nuclear layer (INL) (E) thickness at specified distances from the optic nerve head of the WT and *Emc3* cKO mouse retinas at 12 months of age. The sample size was n = 6 for both WT and cKO mice groups. n = number of independent biological replicates. ANOVA tests were performed for the INL thickness measurement of 3, 6 and 12 months of age. A post-hoc test was performed for the measurement of 12 months of age. * p < 0.05; * * p < 0.01; and * * * p < 0.001. Error bars, SEM.

Immunostaining of retinal cryosections of control and *Emc3* cKO mice with a rod BC marker PKCα antibody revealed a 50% loss of PKCα-positive rod BCs in cKO mice at 12 months of age (Fig 5). To assess pathological changes in *Emc3* cKO mice, we further examined H&E-stained retinal sections of control and *Emc3* cKO retinas at 3, 6 and 12 months of age. The thickness of the ONL and INL in *Emc3* cKO mice was similar to that of controls at 3 months of age (Fig 6A–6C). However, at 6 months of age, the thickness of the INL in the central part of the retina started to decrease compared to that of the controls (Fig 6D–6F). At 12 months of age, while the thickness of the ONL was similar to that of the control retina, the thickness of the INL in *Emc3* cKO retinas was reduced to 55% of that of controls (Fig 6G–6I), indicating loss of cells in the INL. This result was consistent with the PKCα staining results (Fig 5). The INL is occupied by various cell types, such as bipolar cells, amacrine cells, and Muller cells. Given that the loss of *Emc3* occurred only in bipolar cells, the decrease in INL thickness should mainly be due to the loss of BC. No visible defects were observed in the outer segment or inner segment of the rod cells (Fig 6). Consistent with this result, RT-PCR analysis revealed no reduction in rhodopsin mRNA expression in *Emc3* cKO retinas (S4 Fig). Rhodopsin staining also revealed no visible change in the outer segment and inner segment of the rods (S5 Fig).

### Loss of *Emc3* resulted in abnormal staining pattern of mGluR6 and PSD95 in aged mice

**Fig 7:**
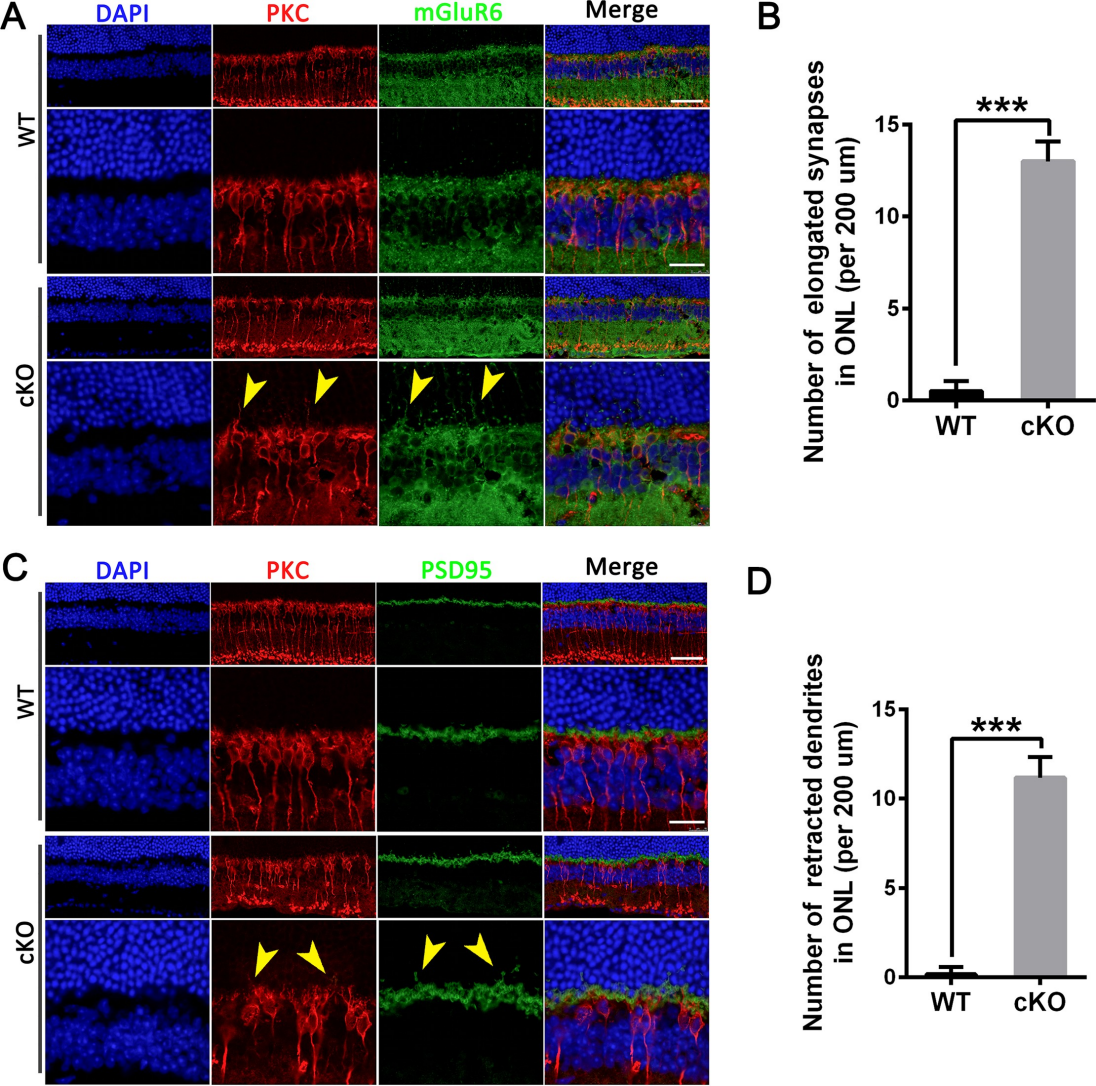
Changed mGluR6 and PSD95 expression patterns of bipolar cells in *Emc3* cKO retinas at 12 months of age. (A) Retinal sections from both WT and cKO mice at 12 months old were labeled with PKCα (red) and mGluR6 (green). Nuclei were counterstained with DAPI (blue). Scale bar, 25 μm. High magnification images are displayed in the lower panel of each image. Scale bar, 10 μm. In control mice, mGluR6-labeled synaptic dendritic processes (green) are confined in the outer plexiform layer (OPL) of control retinas. In contrast, dendritic processes (green) are misplaced (arrows) in the outer nuclear layer (ONL) of *Emc3* cKO retinas. Arrows indicate abnormal dendrite sprouting. (B) Quantification of mGluR6-labeled synaptic dendritic processes in the ONL per 100 μm of sections in the same position. Kolmogorov–Smirnov test indicated that the data set was not normally distributed and a non-parametric Mann–Whitney U test was performed. Z = -2.99; p = 0.003. The number of mGluR6-positive processes increased in 12-month-old cKO mice. The sample size was n = 7 for both WT and cKO mice. n = number of independent biological replicates. * p < 0.05; * * p < 0.01; and * * * p < 0.001. Error bars, SEM. (C) Retinal sections from both WT and cKO mice at 12 months of age were labeled with PKCα (red) and PSD95 (green). Nuclei were counterstained with DAPI (blue). Scale bar, 25 μm. High magnification images are displayed in the lower panel of each image. Scale bar, 10 μm. In control mice, PSD95-labeled synaptic dendritic processes (green) from rods are confined in the outer plexiform layer (OPL) in control retinas. In contrast, rod dendritic processes (green) were retracted into the outer nuclear layer (ONL) in *Emc3* cKO retinas (arrows). Arrows indicate abnormal retracted dendrite sprouting. (D) Quantification of PSD95-labeled synaptic dendritic processes in the ONL per 100 μm of section in the same position. Kolmogorov–Smirnov test indicated that the data set was not normally distributed and a non-parametric Mann–Whitney U test was performed. Z = -2.99; p = 0.003. The number of PSD95-positive processes significantly increased in 12-month-old cKO mice. The sample size was n = 6 for both WT and cKO mice. n = number of independent biological replicates. * p < 0.05; * * p < 0.01; and * * * p < 0.001. Error bars, SEM.

To assess the possible relation between bipolar cell processes and presynaptic inputs, photoreceptor axon terminals were colabelled with mGluR6, PSD95 and PKCα [28]. In the postsynaptic complex of BCs and photoreceptor cells, mGluR6 is localized to dendritic tips and is responsible for glutamate sensing [29–32]. mGluR6 was only observed in the OPL in the retinas of control mice (Fig 7A, upper panels). In contrast, mGluR6-stained punctate structures were observed in the ONL in *Emc3* cKO retinas (Fig 7A, lower panels and 7B). Considering that the mGluR6 receptor is along the PKCα-labeled dendritic fibers in *Emc3* cKO animals, the abnormal postsynaptic processes of mGluR6 labeling might be due to degenerating BCs. In control retinas, bipolar cell dendrites and presynaptic PSD95-labeled rod structures were confined to the OPL (Fig 7C, upper panels). In contrast, in *Emc3* cKO retinas, PSD95-stained puncta retracted back into the ONL, which is a retinal layer normally devoid of synapses (Fig 7C and 7D) at 12 months of age. At 6 months of age, no visible change in the PSD95 staining pattern was observed (S6 Fig). Collectively, these data showed that in *Emc3* cKO mice, BCs exhibit morphological changes at the photoreceptor to bipolar cell synapse at 12 months of age. These results are not consistent with the results from known pre and postsynaptic mutants (detailed information in Discussion). The presence of PSD95-stained puncta in the ONL might be a result of degeneration of BCs and retraction of the connecting afferent rod dendrites [33,34].

### Reactive gliosis in *Emc3* cKO retinas

**Fig 8:**
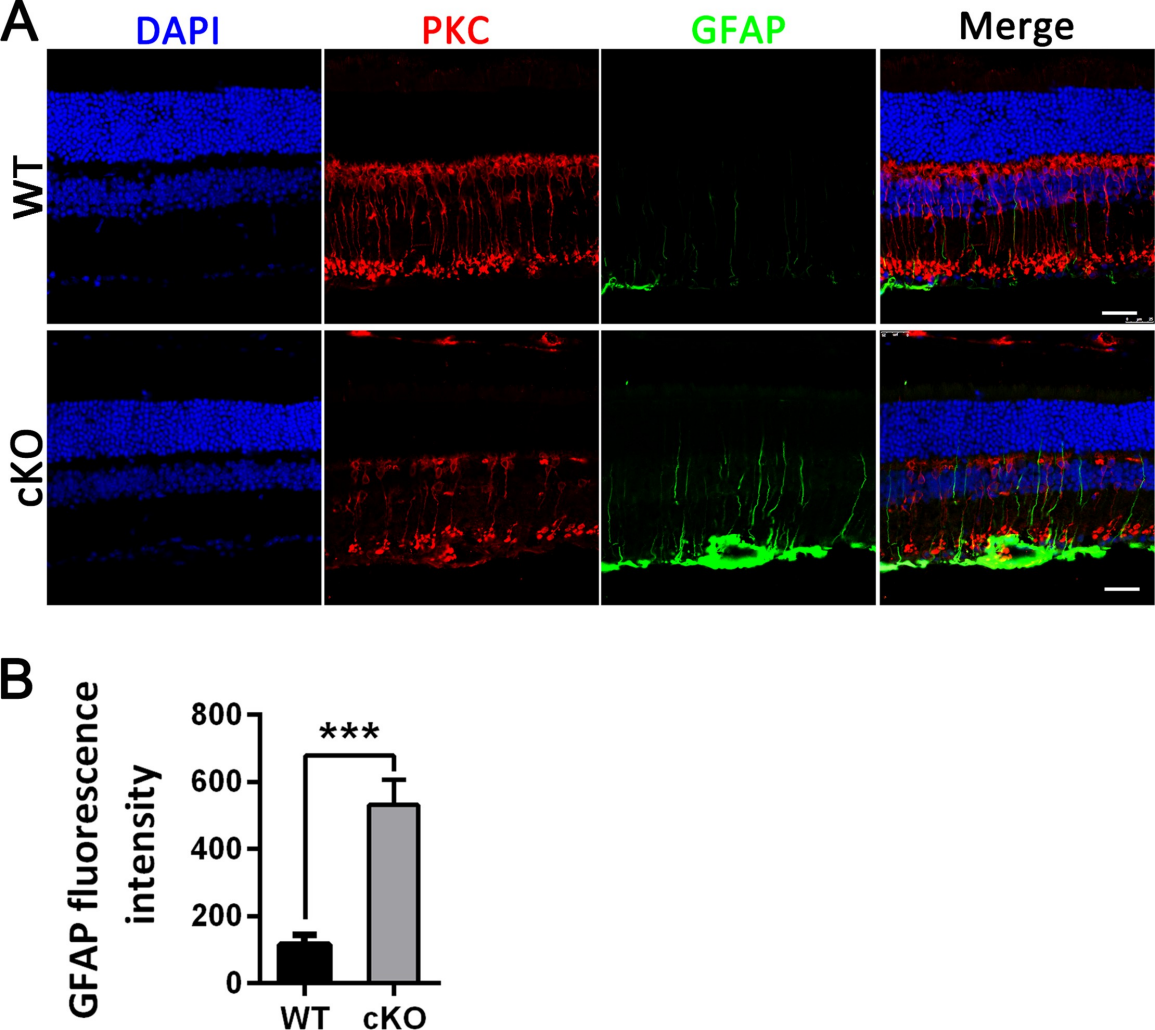
Activation of astrogliosis in *Emc3* cKO retinas. **(A)** Representative immunostained retinal sections of WT and cKO mice stained with PKCα (red) and GFAP (green) at 12 months of age. Nuclei were counterstained with DAPI (blue). Scale bar, 25 μm. (B) Quantification of the fluorescence intensity of GFAP staining of retinal cryosections in WT and *Emc3* cKO retains. Kolmogorov–Smirnov test indicated that the data set was not normally distributed and a non-parametric Mann–Whitney U test was performed. Z = -3.24; p = 0.0003. The intensity of GFAP staining significantly increased in 12-month-old cKO mice. The sample size was n = 5 for both WT and cKO mice. n = number of independent biological replicates. *** p < 0.001. Error bars, SEM.

Generally, inflammation within the central nervous system (CNS) is an important pathological feature of chronic neurodegenerative conditions. Neuroinflammation is characterized by the expression of inflammatory mediators such as cytokines and chemokines as well as glial activation [35]. Microglia and astroglia are two significant contributors to immune responses in the CNS and can secrete cytokines and exert neuroprotective or toxic effects [36–40]. To determine the retinal injury status in *Emc3* cKO mice, we assessed glial activation in the retina. Muller cells are important for maintaining the structural and functional stability of retinal cells. Activated Muller glia were also detected in 12-month-old cKO retinas by immunostaining with an antibody against GFAP (Fig 8A), which is an intermediate filament protein that is a major component of astrocytes. Compared to minimal staining for GFAP in controls and *Emc3* cKO mice at 6 months of age (S7 Fig), intense staining for GFAP was observed throughout the retina of *Emc3* cKO mice at 12 months of age (Fig 8A and 8B), suggesting a severe Muller glial cell response to retinal damage or stress. This may ultimately result in loss of BCs.

## Discussion

The EMC is critical for the stable expression of multipass transmembrane proteins such as rhodopsin, and its loss causes retinal degeneration in *Drosophila* and mice [18, 19]. However, the specific function of *Emc3* in BCs has not been explored. In this study, we explored the roles of *Emc3* in BCs using a BC-specific knockout mouse model: *Emc3* cKO mice. *Emc3* cKO mice exhibited degeneration of BCs, and ERG test revealed that the amplitudes of the scotopic and photopic b-waves were markedly decreased in *Emc3* cKO mice at 12 months of age (Figs 2 and 3), while photoreceptor function appeared intact. BC degeneration subsequently led to severe gliosis (Fig 8) and death of BCs (Fig 5), similar to the phenotypes of the *Tmem30a* BC deletion mouse model that was previously described [41]. Mechanistically, loss of *Emc3* might disrupt the synthesis and transport of important membrane proteins in BCs. The exact underlying molecular mechanisms warrant further investigation.

The reduced amplitude of the b-wave measured by ERG analysis in *Emc3* cKO retinas could be due to signaling defects in the mutant BCs. This is also possibly due to there being fewer BCs in cKO mice. The amplitude of the b-wave of BCs in *Emc3* cKO retinas was reduced by approximately half of that of control littermates at 12 months of age. In contrast to the rapid degeneration of rod cells deficient in *Emc3*, death of BCs progressed slowly (Fig 6). One possible explanation is that in *Emc3* BC cKO mice, the course of BC death is chronic compared to the rapid death of rods, which has high demands for energy and protein synthesis in light sensing cilia structures. In our *Emc3* cKO model, deletion of *Emc3* in BCs directly affects the function of the ER complex in 75% of BCs. Membrane proteins with multiple transmembrane domains are more likely affected in the absence of *Emc3*. Therefore, the physiological function of BCs can be progressively affected. At 12 months of age, the amplitude of the scotopic ERG b-wave is reduced by 50%, and the amplitude of the photopic ERG b-wave is reduced by 30% (Figs 3 and 4). As the mice age, the phenotypes might become more severe.

In previously reported mouse models, different retinal morphological changes were observed in presynaptic and postsynaptic mutant models. The synaptic ribbons in rod photoreceptors are abnormal or absent, and the dendrites of BCs extend into the outer nuclear layer in *Cacnb2*, *Basson* and *nob2* (Cacna1f^nob2^) mutants [42–44]. Ectopic synapses were observed in bassoon KO mice [43]. In contrast, retinal structure is normal in four postsynaptic mutants: *Grm6*^*Tm1Nak*^ [45, 46], *Grm6*^*nob4*^ [47], *Gnao1*^*tm1Lbi*^ [48] and Nyx^nob^ [49, 50]. However, abnormal OPL morphology was observed in Gβ5 knockout mice, which was probably due to altered expression of the long isoform of Gβ5 in photoreceptors rather than a change in the short isoform in BCs [51]. In our *Emc3* cKO mouse model, elongation of mGluR6-stained processes was observed in the ONL, and ecotopic expression of the PSD95 puncta was also observed (Fig 7). These results are not consistent with the results from known *Grm6*^*Tm1Nak*^ [45, 46], *Grm6*^*nob4*^ [47], *Gnao1*^*tm1Lbi*^ [48] and Nyx^nob^ [49, 50]. Furthermore, a thinner INL was observed at 12 months of age (Fig 6). Given the important roles of the EMC in the proper synthesis and transport of membrane proteins in the ER, this change might be due to changed expression/localization of various membrane proteins in *Emc3* mutant BCs. The exact underlying molecular mechanisms warrant further investigation.

In summary, our data demonstrated the importance of *Emc3* in the long-term survival of retinal BCs.

## Supporting information

S1 Fig *Emc3* is expressed in the retinal BCs. Cryosection of retinal sections form 4 weeks old mice were double-labeled with EMC3 and RHO antibodies. EMC3 is strongly expressed in the outer segment of the rod cells. EMC3 is also expressed in bipolar cells. Nuclei were counterstained with 4′,6-diamidino-2-phen (DAPI). Scale bar, 25 μm.
(PDF)

S2 Fig Conditional deletion of *Emc3* with Pcp2-Cre. Design of the *Emc3* conditional knockout allele (cKO) is shown. Critical exon 2 is flanked by two loxP sites. The *Emc3* floxed allele (*Emc3*^*fl*^) was crossed to Pcp2-Cre to generate tissue-specific knockout models.
(PDF)

S3 Fig *Emc3* cKO mice were genotyped by PCR. Genomic DNA from mouse tail lysates of control (WT), heterozygous (het), and *Emc3* cKO mice was amplified using the primer pair EMC3-Seq-F1 and EMC3-Seq-R1. The floxed allele yielded a PCR product of 287 bp, while the wild-type allele yielded a PCR product of 247 bp. Cre was genotyped using Cre-F and Cre-R. A product of 350 bp can be amplified in Cre-positive mice.
(PDF)

S4 Fig RT-PCR analysis revealed no changes in rhodopsin mRNA levels in *Emc3* cKO retinas. A test was performed. N = 4 for both controls and *Emc3* cKO mice. ns, no statistical significance.
(PDF)

S5 Fig No changes were observed in the outer segment (OS) or inner segment (IS) of *Emc3* cKO retinas. Retinal cryosections from controls and *Emc3* cKO (Emc3-Pcp2-Mut) mice at 12 months of age were labeled with the OS marker rhodopsin (upper panel) and the IS marker Na-K ATPase (lower panel) (green). Compared to controls, no changes were observed in OS and IS in *Emc3* cKO mice. Nuclei were counterstained with 4′,6-diamidino-2-phen (DAPI). Scale bar, 20 μm.
(PDF)

S6 Fig PSD95 staining of *Emc3* cKO retinas at 6 months of age. Retinal sections from both WT and cKO mice at 6 months of age were labeled with PKCα (red) and PSD95 (green). Nuclei were counterstained with DAPI (blue). Scale bar, 25 μm. In both control and *Emc3* cKO mice, PSD95-labeled synaptic dendritic processes (green) from rods are confined in the outer plexiform layer (OPL). Compared to control mice, no difference in the pattern of PSD95 staining was observed in 6-month-old cKO mice. Scale bar, 25 μm.
(PDF)

S7 Fig GFAP staining in *Emc3* cKO retinas at 6 months of age. Representative immunostained retinal sections of WT and cKO mice stained with PKCα (red) and GFAP (green) at 6 months of age. Nuclei were counterstained with DAPI (blue). Scale bar, 25 μm. Compared to control mice, no difference in the intensity of GFAP staining was observed in 6-month-old cKO mice. Scale bar, 25 μm.
(PDF)

S1 Table Primers used in this study. (PDF)

S2 Table Number of mice used in each test. (PDF)

S1 File (PDF)
