# Supplementary material for: Loss of the ER membrane protein complex subunit Emc3 leads to retinal bipolar cell degeneration in aged mice
Source: PLoS One. 2020 Sep 4;15(9):e0238435. doi: 10.1371/journal.pone.0238435 (PMC7473584; doi:10.1371/journal.pone.0238435)
Supplement: S1 Fig — Cryosection of retinal sections form 4 weeks old mice were double-labeled with EMC3 and RHO antibodies. EMC3 is strongly expressed in the outer segment of the rod cells. EMC3 is also expressed in bipolar cells. Nuclei were counterstained with 4′,6-diamidino-2-phen (DAPI). Scale bar, 25 μm. (PDF) [file pone.0238435.s001.pdf]

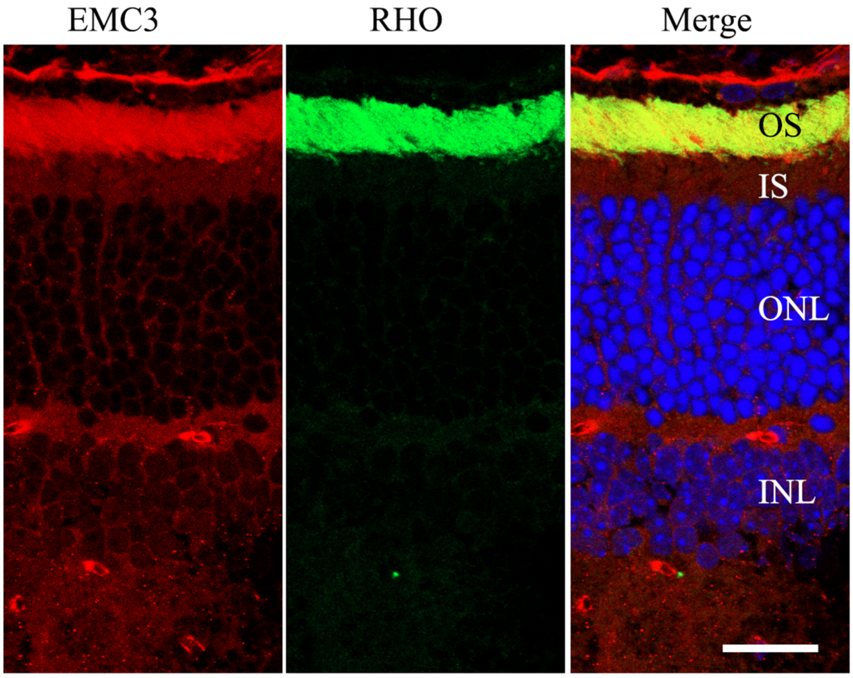

Figure S1. *Emc3* is expressed in the retinal BCs. Cryosection of retinal sections form 4 weeks old mice were double-labeled with EMC3 and RHO antibodies. EMC3 is strongly expressed in the outer segment of the rod cells. EMC3 is also expressed in bipolar cells. OS, outer segment; IS, inner segment. Nuclei were counterstained with 4′,6-diamidino-2-phen (DAPI). Scale bar, 25 μm.
