# Supplementary material for: Loss of the ER membrane protein complex subunit Emc3 leads to retinal bipolar cell degeneration in aged mice
Source: PLoS One. 2020 Sep 4;15(9):e0238435. doi: 10.1371/journal.pone.0238435 (PMC7473584; doi:10.1371/journal.pone.0238435)
Supplement: S2 Fig — Design of the Emc3 conditional knockout allele (cKO) is shown. Critical exon 2 is flanked by two loxP sites. The Emc3 floxed allele (Emc3fl) was crossed to Pcp2-Cre to generate tissue-specific knockout models. (PDF) [file pone.0238435.s002.pdf]

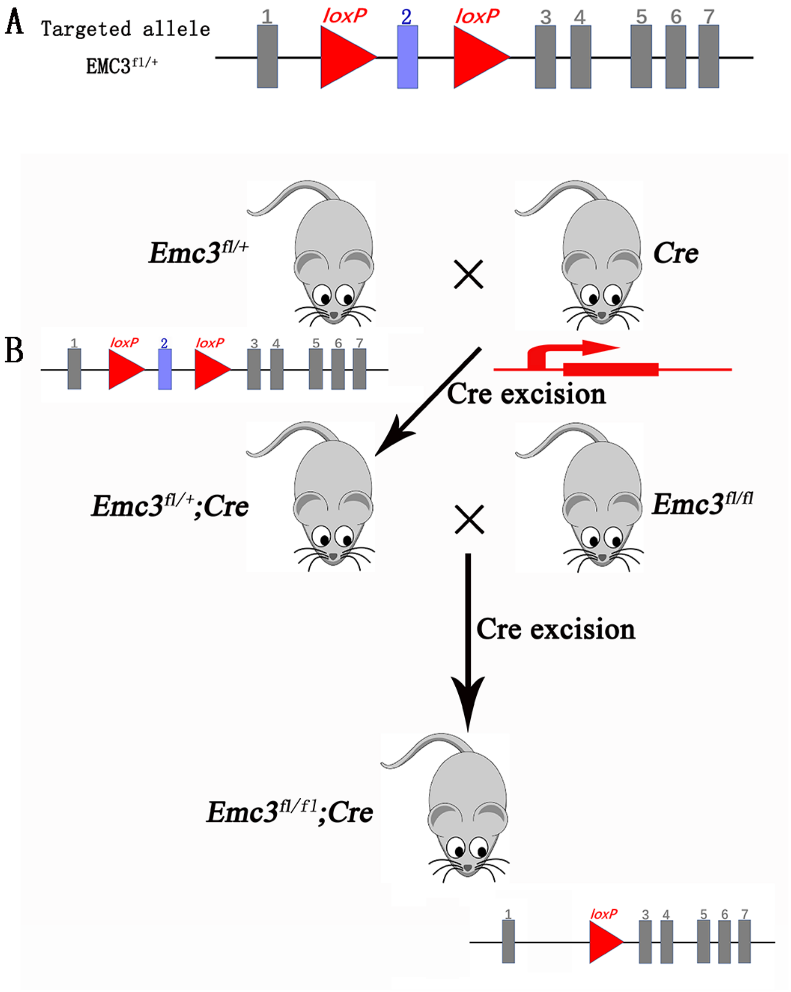

Figure S2. Conditional deletion of *Emc3* with Pcp2-Cre. Design of the *Emc3* conditional knockout allele (cko) is shown. The critical exon 2 is flanked by two loxP sites. The *Emc3* floxed allele (*Emc3*^{fl}) was crossed to Pcp2-Cre to generate tissue specific knockout models.
