# Supplementary material for: Loss of the ER membrane protein complex subunit Emc3 leads to retinal bipolar cell degeneration in aged mice
Source: PLoS One. 2020 Sep 4;15(9):e0238435. doi: 10.1371/journal.pone.0238435 (PMC7473584; doi:10.1371/journal.pone.0238435)
Supplement: S3 Fig — Genomic DNA from mouse tail lysates of control (WT), heterozygous (het), and Emc3 cKO mice was amplified using the primer pair EMC3-Seq-F1 and EMC3-Seq-R1. The floxed allele yielded a PCR product of 287 bp, while the wild-type allele yielded a PCR product of 247 bp. Cre was genotyped using Cre-F and Cre-R. A product of 350 bp can be amplified in Cre-positive mice. (PDF) [file pone.0238435.s003.pdf]

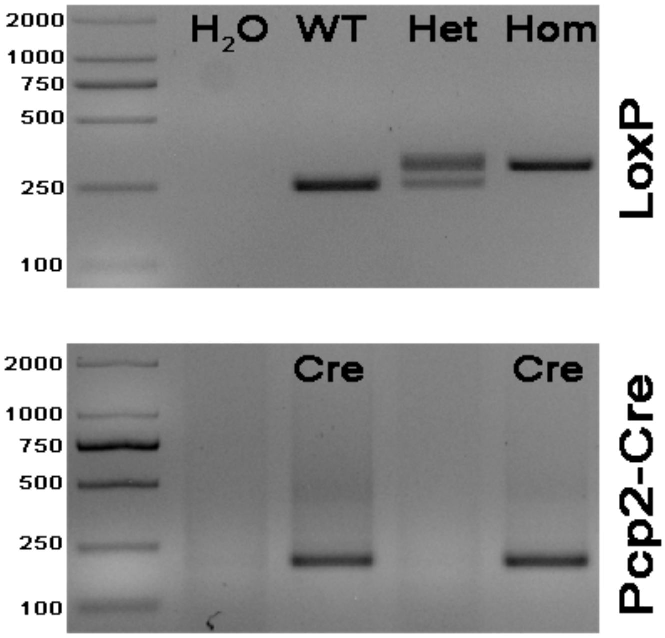

Fig. S3. Genotyping *Emc3* cKO mice by PCR method. Genomic DNA from mouse-tail lysate of control (WT), heterozygous (het), and *Emc3* cKO mice were amplified using primer pair Emc3-F1 and Emc3-R1. Floxed allele yields a PCR product of 287 bp, while wildtype allele yields a PCR product of 247 bp. Cre was genotyped using Cre-F and Cre-R. A product of 350 bp can be amplified in Cre positive mice.
