# Supplementary material for: Loss of the ER membrane protein complex subunit Emc3 leads to retinal bipolar cell degeneration in aged mice
Source: PLoS One. 2020 Sep 4;15(9):e0238435. doi: 10.1371/journal.pone.0238435 (PMC7473584; doi:10.1371/journal.pone.0238435)
Supplement: S4 Fig — A test was performed. N = 4 for both controls and Emc3 cKO mice. ns, no statistical significance. (PDF) [file pone.0238435.s004.pdf]

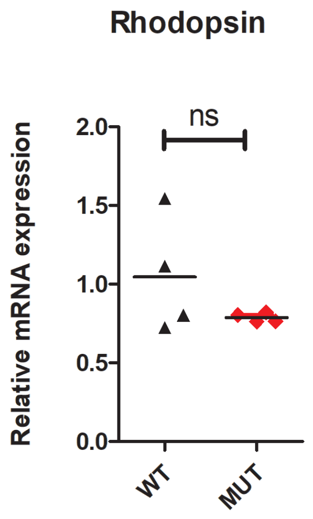

Fig. S4. RT-PCR analysis revealed no changes were observed in Rhodopsin mRNA level in *Emc3* cKO retinas. A test was performed. N=4 for both controls and *Emc3* cKO. ns, no statistical significance.
