# Supplementary material for: Loss of the ER membrane protein complex subunit Emc3 leads to retinal bipolar cell degeneration in aged mice
Source: PLoS One. 2020 Sep 4;15(9):e0238435. doi: 10.1371/journal.pone.0238435 (PMC7473584; doi:10.1371/journal.pone.0238435)
Supplement: S5 Fig — Retinal cryosections from controls and Emc3 cKO (Emc3-Pcp2-Mut) mice at 12 months of age were labeled with the OS marker rhodopsin (upper panel) and the IS marker Na-K ATPase (lower panel) (green). Compared to controls, no changes were observed in OS and IS in Emc3 cKO mice. Nuclei were counterstained with 4′,6-diamidino-2-phen (DAPI). Scale bar, 20 μm. (PDF) [file pone.0238435.s005.pdf]

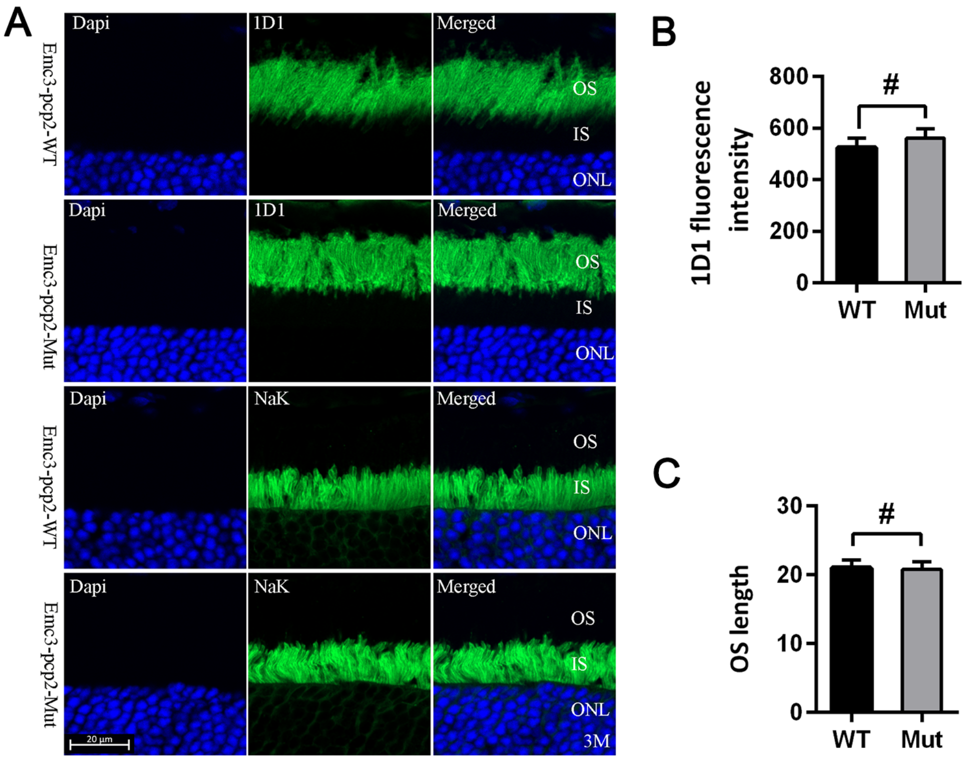


**Fig. S5. No changes were observed in outer segment (OS) and inner segment (IS) of *Emc3* cKO retinas**. Retina cryosections from controls and *Emc3* cKO (Emc3-Pcp2-Mut) at 12 months of age were labeled with OS maker Rhodopsin (upper panel) and IS marker Na-K ATPase (lower panel) (green). Compare to controls, no changes were observed in OS and IS in *Emc3* cKO mice. Nuclei were counterstained with 4′,6-diamidino-2-phen (DAPI). Scale bar, 20 μm.
