# Supplementary material for: Loss of the ER membrane protein complex subunit Emc3 leads to retinal bipolar cell degeneration in aged mice
Source: PLoS One. 2020 Sep 4;15(9):e0238435. doi: 10.1371/journal.pone.0238435 (PMC7473584; doi:10.1371/journal.pone.0238435)
Supplement: S6 Fig — Retinal sections from both WT and cKO mice at 6 months of age were labeled with PKCα (red) and PSD95 (green). Nuclei were counterstained with DAPI (blue). Scale bar, 25 μm. In both control and Emc3 cKO mice, PSD95-labeled synaptic dendritic processes (green) from rods are confined in the outer plexiform layer (OPL). Compared to control mice, no difference in the pattern of PSD95 staining was observed in 6-month-old cKO mice. Scale bar, 25 μm. (PDF) [file pone.0238435.s006.pdf]

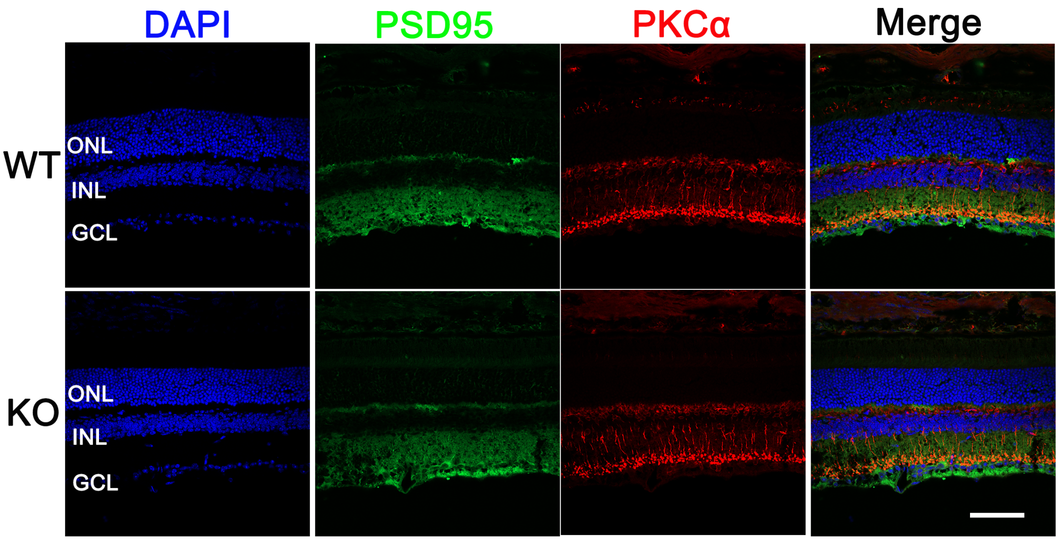

Fig. S6. PSD95 staining of rod bipolar cells in *Emc3* cKO retinas. Retinal sections from both WT and cKO mice at 6-month old were labeled with PKCα (red) and PSD95 (green). Nuclei were counter-stained with DAPI (blue). Scale bar, 25 μm. In both control and *Emc3* cKO mice, PSD95 labeled synaptic dendritic processes (green) from rods are confined in the outer plexiform layer (OPL). Compared to control mice, no difference in the pattern of PSD95 staining was observed in 6-month-old cKO mice. Scale bar, 25 μm.
