# Supplementary material for: Loss of the ER membrane protein complex subunit Emc3 leads to retinal bipolar cell degeneration in aged mice
Source: PLoS One. 2020 Sep 4;15(9):e0238435. doi: 10.1371/journal.pone.0238435 (PMC7473584; doi:10.1371/journal.pone.0238435)
Supplement: S7 Fig — Representative immunostained retinal sections of WT and cKO mice stained with PKCα (red) and GFAP (green) at 6 months of age. Nuclei were counterstained with DAPI (blue). Scale bar, 25 μm. Compared to control mice, no difference in the intensity of GFAP staining was observed in 6-month-old cKO mice. Scale bar, 25 μm. (PDF) [file pone.0238435.s007.pdf]

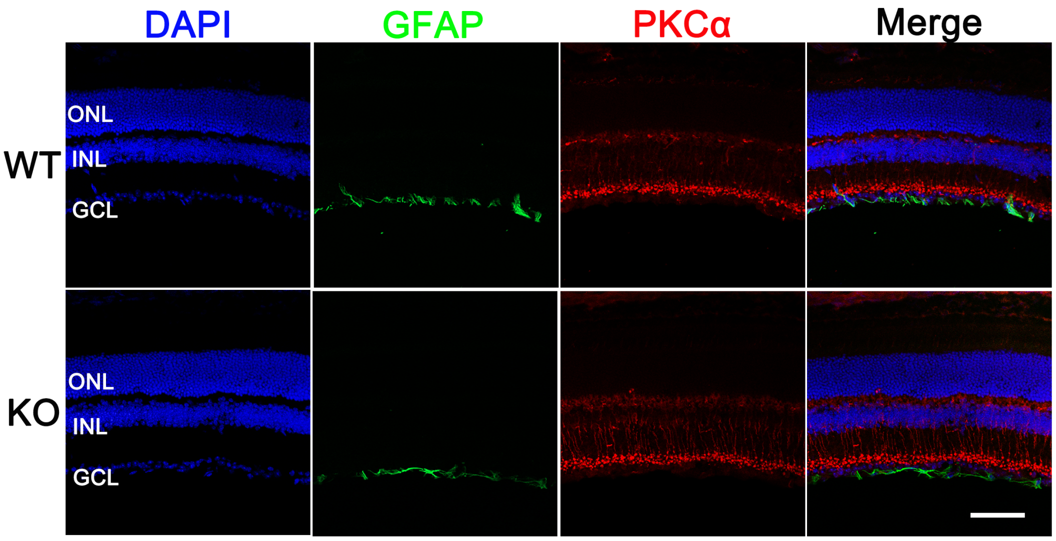

Figure S7. GFAP staining in *Emc3* cKO retinas. Representative immunostained retinal sections of WT and cKO mice stained with PKCα (red) and GFAP (green) at 6 months of age. Nuclei were counter-stained with DAPI (blue). Scale bar, 25 µm. Compared to control mice, no difference in the intensity of GFAP staining was observed in 6-month-old cKO mice. Scale bar, 25 µm.
