# Supplementary material for: Loss of the ER membrane protein complex subunit Emc3 leads to retinal bipolar cell degeneration in aged mice
Source: PLoS One. 2020 Sep 4;15(9):e0238435. doi: 10.1371/journal.pone.0238435 (PMC7473584; doi:10.1371/journal.pone.0238435)
Supplement: S1 Table — (PDF) [file pone.0238435.s008.pdf]

Table S1. Primers used in this study

Primer name	Primer sequence
Emc3-F1	5’-TGTCTCCCGTCAAATCCAGAAAGG-3’
Emc3-R1	5’-ATGTGAAACGCAAAGAAAGCAAGC-3’
Cre-F	5’-ATTTGCCTACATTACCGGTC–3’
Cre-R	5’-ATCAACGTTTTCTTTTCGG-3’
Emc3-RT-F	5’-TTTTCCATTTTCCCTGAGGA-3’
Emc3-RT-R	5’-TAGGCATGATCCGCCACTAC-3’
Rhodopsin-RT-F	5’-AGGCAGAGAAGGAAGTCACC-3’
Rhodopsin-RT-R	5’-GCAAAGAAAGCTGGCAGAGT-3’
