# Supplementary material for: Loss of the ER membrane protein complex subunit Emc3 leads to retinal bipolar cell degeneration in aged mice
Source: PLoS One. 2020 Sep 4;15(9):e0238435. doi: 10.1371/journal.pone.0238435 (PMC7473584; doi:10.1371/journal.pone.0238435)
Supplement: S2 Table — (PDF) [file pone.0238435.s009.pdf]

Table S2. Number of mice used in each test

ID	Test	Number of mice used	Figure number
1	Tomato reporter assay	N=4 for both WT and cKO	Fig. 1A
2	RT-PCR study for Emc3 expression	N=4 for both WT and cKO	Fig. 1B
3	ERG test at 6 month of age	N=4 for both WT and cKO	Fig. 2, 4
4	ERG test at 12 month of age	N=4 for both WT and cKO	Fig. 3, 4
5	Immunostaining analysis	N=6 for both WT and cKO	Fig. 5
6	H&E staining at 3 months of age	N=4 for both WT and cKO	Fig. 6
7	H&E staining at 6 months of age	N=4 for both WT and cKO	Fig. 6
8	H&E staining at 12 months of age	N=6 for both WT and cKO	Fig. 6
9	mGluR6 staining	N=7 for both WT and cKO	Fig. 7
10	PSD95 staining	N=6 for both WT and cKO	Fig. 7
11	GFAP staining	N=5 for both WT and cKO	Fig. 8

WT, wildtype; cKO, *Emc3* BC-knockout mice.
