# Supplementary figures and images for: Loss of the ER membrane protein complex subunit Emc3 leads to retinal bipolar cell degeneration in aged mice
Source: PLoS One. 2020 Sep 4;15(9):e0238435. doi: 10.1371/journal.pone.0238435 (PMC7473584; doi:10.1371/journal.pone.0238435)

Uncropped imaged for figure 1B and figure S2.

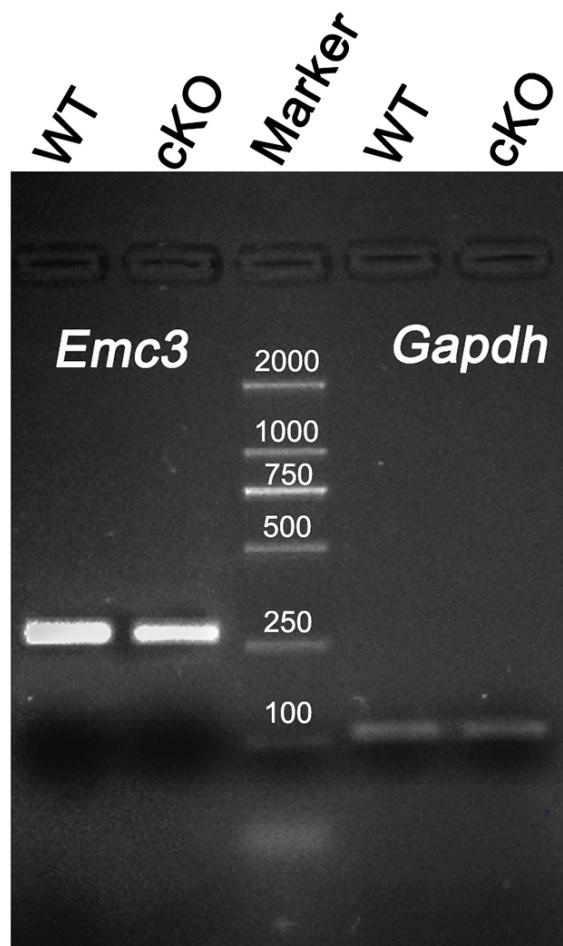

Uncropped images for Figure 1B

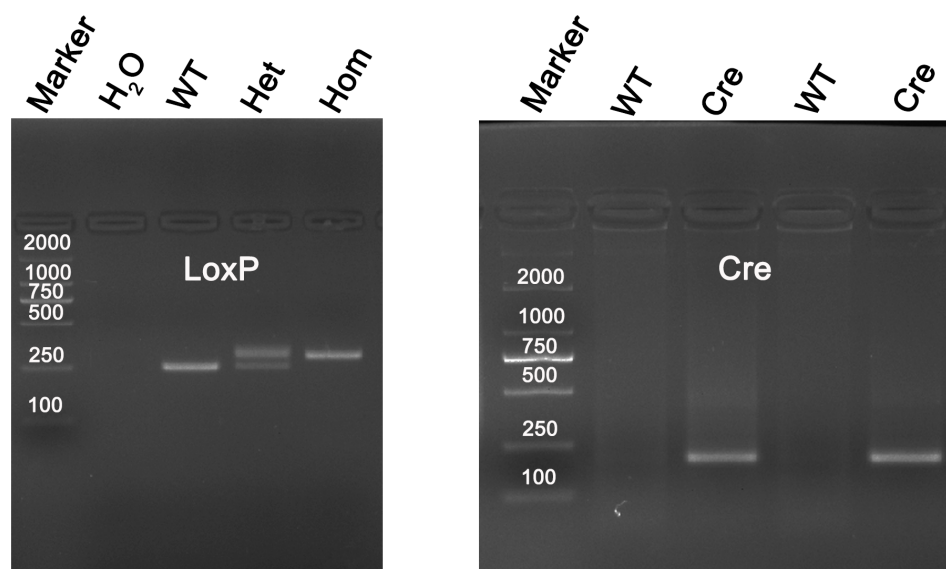

Figure S2B

Supplement: S1 File — (PDF) [file pone.0238435.s010.pdf]
